# The Predisposing Factor in Dental Disorders

**Published:** 1899-05

**Authors:** Edward C. Kirk

**Affiliations:** Philadelphia


### International Dental Journal.

Vol. XX. May, 1899. No. 5.

### Original Communications.[1]

### THE PREDISPOSING FACTOR IN DENTAL DISORDERS.[2]

BY EDWARD C. KIRK, D.D.S., PHILADELPHIA.

In studying the morbid phenomena found in the oral cavity there has been a tendency among members of the dental profession to view the problem in a somewhat restricted manner; to overlook the broader aspects of the subject presented by its relations to the whole organism, and the still broader significance of its biological relations. It is this factor in the development of a specialism in any department of human inquiry which is restrictive of the highest rate of advancement, and which should therefore be carefully guarded against so far as that may be possible.

Happily, the stimulus which a few investigators have given to the spirit of original research in dentistry is bearing good fruit, and the practical advantages of scientific over purely empirical methods of inquiry are making an impress upon our practice which is placing it more and more upon a rational basis.

The result is clearly seen in our literature, especially as it reflects the views now held with respect to dental pathology in com-

[1] The editor and publishers are not responsible for the views of authors of papers published in this department, nor for any claim to novelty, or otherwise, that may be made by them. No papers will be received for this department that have appeared in any other journal published in the country.

[2] Read before the Alumni Association of the Boston Dental College, February 8, 1899.

parison with the narrower views of former years. The investigations of cytologists have opened up vistas of knowledge giving us a comprehension of cell-development and function which has furnished us a firm basis upon which to build a rational understanding of physiology and pathology, and which is rapidly bringing these important branches of study into line with the exact sciences by resolving nearly all of their phenomena into problems of physics and chemistry.

Dentistry has not only felt the influence of this progressive movement, but has contributed in no small degree to its growth. The results of original investigation in the domain of dental histology and dental pathology are monuments of which we may well be proud; yet it is research within the comparatively narrow limits of a specialty, as already noted. I would not be understood as maintaining an attitude of adverse criticism in stating the case as I have, but rather as indicating the desirability, if not the necessity, for a broader scope to our horizon in order that our professional grasp may be more comprehensive.

I can perhaps best illustrate the view-point which I am endeavoring to present to you by asking your consideration of certain predisposing factors in dental pathological conditions which directly bear upon the necessity for a broad study of principles rather than precepts in our specialty.

The study of the human organism, both in health and in disease, has quickly resolved itself into a study of the development and vital phenomena of the cellular elements of which it is made. Not until we have attained a fair understanding of cell-development and function is it possible to intelligently comprehend the vital phenomena of the organism as a whole, and especially these aberrations from normal standards of cell-function which we designate disease.

At the very outset of our investigations of cell-function we are met by an apparently insurmountable difficulty,—namely, the phenomenon of vitality, of life. All attempts at a comprehension, much less an explanation, of it are baffled by the fact itself. Yet when viewed as philosophic abstractions the same difficulty confronts us when we study matter or force in any of their manifestations to our senses; nevertheless, we understand enough of the properties of matter and of force to make them in many ways subservient to our needs, and so in a practical sense we comprehend them. So also in a limited sense we may comprehend vitality as a function or attribute of protoplasm, and for purposes of physiological study regard it as a form of energy the product of chemical changes associated with cell-metabolism.

Physiologists teach us that, "broadly speaking, the animal body is a machine for converting potential energy into active energy. The potential energy is supplied by food, this the metabolism of the body converts into the actual energy of heat and mechanical labor," and as what is true of the mass is also true of its parts, the same view is true of the cellular elements of which the body is composed. In fact, it is through the metabolic agency of the cellular protoplasm that the potential energy of food is rendered kinetic. It is in connection with this transmutation of energy that vitality is made manifest, and with the metabolic process it bears a direct relation quantitatively.

With these propositions before us it is not difficult to realize that a maximum cell vitality is attainable when the supply of food potential to the cell is harmoniously adjusted to its metabolic capacity, and that any alteration of its nutritive balance must necessarily work a corresponding decrease in vital potential with an attendant alteration of cell-function.

It is these alterations of nutritive balance in the cell and attendant disturbance of function that constitute disease. The causes which lead to disturbances of cell-metabolism and diminution of vitality may arise within the organism itself or be introduced from without, and, further, the disturbance may be local or it may be general.

When the disturbing factor is a specific irritant introduced from without we regard it as the exciting or active cause of disease; on the other hand, when the disturbance is preceded by an interference with cell vitality due to faulty metabolism we classify the latter as a predisposing cause.

It is evident that the factor of vital potential is a safeguard against invasion by the multitudes of lower organic forms which are pathogenic to the human organism and which are ever-present factors of its environment. Vitality is the defensive force of the organism against pathological attack, and is, in general terms, effective in proportion to its degree of potentiality.

It is not my purpose this evening to consider the invading enemy so much as the defensive forces of the organism, and to ask your consideration of some of the conditions which lead to a weakening of the vital potential and the bearing of this upon certain oral lesions in which we are all presumably interested.

Time will not permit of the study of that large class of acute inflammatory processes the result of specific infections by microorganisms of the more active and virulent type, nor a study of the factor of immunity in relation to them; the most that I can hope to do is to briefly sketch some of the predisposing factors which are apparently closely related to the inflammatory disorders affecting the retentive apparatus of the teeth.

Attention to the disease designated by Black as phagedenic pericementitis was actively revived by the announcement of Peirce, in 1892, that its etiology was to be found in its connection with the gouty diathesis, a conclusion which he reached in view of the results of certain analyses showing uric acid to be a constituent of the concretions found upon the roots of teeth affected by the disorder in quesiton. That Peirce's view contained a large element of truth is demonstrably true; that it was the whole truth even its author did not claim; but by the direction which his observations gave to the thought of investigators we are gradually unravelling the entanglement of mystery which has for so long involved the study of this disorder.

Uric acid, as is well known, is one of the waste products of cell-metabolism; it represents in part the excess of nitrogen thrown out in the process of tissue metamorphosis, and is, within certain limits, one of the normal products of nutrition. Like all other waste products of vital action, it is poisonous to the organism that produces it if not normally eliminated or if produced in excess. Haig places the ratio of uric acid formation to that of urea from 1 to 33 to 1 to 35 as the normal, and when in excess of that quantity regards it as productive of disturbance in the economy acting as an irritant poison.

The effects of chronic uric acid poisoning have been pretty fully studied, its action upon the nervous system in the production of migraine, various neuroses, neurasthenia, indigestion, catarrhal affections, gouty manifestations, nephritis, and general malnutrition are all well known. It is, however, not only directly pathogenic by reason of its irritative effect upon the tissues, but indirectly so by the power which it has to diminish the bactericidal activity of the blood plasma, as shown by the experiments of Pansini and Calabrese (1894). These investigators found that the addition of uric acid to blood overcame the activity of the alexins or defensive proteids produced by the white blood-cells. An organism saturated with an excess of uric acid becomes more vulnerable to invasion by disease-producing bacteria.

While a *rôle* of pre-eminent importance has been assigned, no doubt justly, to uric acid in disorders consequent upon general malnutrition, investigation of the phenomena incident to uricacidæmia has developed the important discovery that uric acid is by no means wholly responsible for all of the pathological results which have been attributed to it. It has been shown that a related group of substances, the so-called alloxuric bodies, are chargeable in some instances, to even a greater degree than uric acid, with the production of pathological manifestations in the economy. The alloxuric bodies are, like their congener uric acid, waste products of nitrogenous metabolism.

An interesting problem presents itself in attempting to explain the relation of these waste products to the disorders with which their existence in the blood is associated,—*e.g.*, gout, malnutrition, etc.

Alexander Haig and his followers take the view that uric acid in excess is due to faulty dietetics, insufficient exercise, etc., and that the waste products are taken up by the blood and act as chemical or even mechanical irritants to the tissues fed by the blood-stream, the tissues responding pathologically in the order of their relative resistance to the morbid influence. More recently this view has been combated. Horbaczewski and others have shown that uric acid is produced by the splitting up of the nuclein of the white blood-cells, and that an abnormal increase of these cells is coincident with an increase in the production of uric acid.

It has further been demonstrated that irritation of the vasomotor sympathetic is followed by a leucocytosis, hence the conclusion is that excessive production of the alloxuric bodies is the result of a neurosis; not the cause of the disease, but its effect.

Neusser has, however, shown that the alloxuric bodies are irritant to the sympathetic nervous system, and it would therefore seem, in considering the operations of these substances as disease factors, that we have to do with a so-called vicious circle, regardless of the initial cause; if we accept the facts as stated, then irritation of the vasomotor sympathetic induces increased formation of leucocytes, which causes excessive production of uric acid products, which in turn bring about increased irritation of the sympathetic and repetition of the steps of the process indefinitely.

Aside from the experimental studies by Peirce, our grounds for considering these matters as related to inflammatory conditions of the peridental membrane and its surrounding tissues are those furnished by the clinical study of cases.

The constant association of phagedenic pericementitis with nearly all well-marked cases of lithæmia must now be regarded as something more than a mere coincidence. Much antagonism to the so-called "uric acid theory of pyorrhœa alveolaris" has grown out of the assumption that all cases of pyorrhœa alveolaris were believed to be due to excess of uric acid in the blood. No such sweeping claim was ever made by the supporters of the theory. It must be generally recognized by all who have carefully observed the clinical aspects of the disorder that necrotic inflammation of the retentive apparatus of the tooth is induced by any cause that will depress the vital potential of its elements to a point where bacterial invasion becomes a factor in the process. But that the systemic condition known as lithæmia, or uricacidæmia, by which is meant a constant over-production and imperfect elimination of the waste products of nitrogenous metabolism, is a most potent factor in the reduction of vital resistance in the tissues alluded to is also equally evident.

I have admitted the bacterial element as a possible exciting cause, even though it has not yet been demonstrated, not because I am wholly convinced that it is a necessary factor in explaining the etiology of the disorder, but because in the later stages of the process it is necessarily present, and then undoubtedly plays a *rôle* of more or less importance. The tendency to ascribe all inflammatory conditions to bacterial irritation is as faulty, in view of the facts, as to ascribe all forms of pyorrhœa to uric acid poisoning.

We should not overlook the fact, however, that these waste products of nitrogenous metabolism are irritant and even poisonous to the tissues when present in sufficient quantity or when their action is prolonged indefinitely, and there would seem to be no sound reason why, under favorable circumstances, their function as predisposing factors should not become resolved into the exciting cause of the disease process under consideration.

Their action in inducing certain forms of nephritis has been pretty clearly demonstrated, and likewise their etiological relation to arterial atheroma. They act as protoplasmic poisons, and in so doing they differ in nowise excepting as to degree of virulency from the poisonous excreta of bacteria in their effect upon the tissue elements.

In our study of the progressive necrotic inflammatory process known as phagedenic pericementitis it would seem that our conceptions of its nature, especially in its earlier manifestations, have been modified, if not hampered, by the definitions of inflammation arranged for us from the point of view of the bacterial pathologist. The presence of specific bacterial exciters of the inflammatory process seem in many cases to have been assumed as necessary to the inflammatory action. But the scientific method requires that a definition be fitted to the phenomena, not the phenomena to the definition. Pathology furnishes many examples of tissual reaction towards irritant substances other than bacterial excreta which in their essential characteristics are entirely analogous to so-called true inflammation, and which should be included in any comprehensive definition of that process.

It is, in the opinion of your essayist, to the class of non-bacterial inflammatory tissue reactions that phagedenic pericementitis in its earlier stages belongs, and that the toxic irritant is the group of alloxuric bodies which, as the result of faulty metabolism, find their way into the blood-stream and thence to the membranous investment of the tooth, that are the active cause of degeneration of the tissue in question, and, should the irritative influence be of sufficient intensity as related to the vital resistance of the elements of the membrane, may and do cause its molecular necrosis with attendant inflammatory reaction.

I have elsewhere drawn attention to the acute and chronic forms of the disorder as clinically manifested in pericemental abscess and chronic pyorrhœa alveolaris respectively.

It will be seen that in the view here presented the disease has a strongly indicated constitutional origin, certainly so far as its predisposing cause is concerned, so that, if it is to be successfully combated, the systemic vice must first of all be eliminated as a controlling factor.

This phase of the treatment naturally falls within the sphere of the physician, yet in my own experience I have found but a small minority of physicians who have given the subject more than superficial consideration. For that reason it may not be out of place to refer to a few of the clinical phenomena which these cases present, in order that they may help us not only to recognize the condition, but to indicate to the medical adviser the lines along which his efforts are needed.

A constant symptom which is or has been present as the earliest manifestation of error in the nutritional process is constipation. Nearly all patients will admit that they were in good health and had no evidences of the dental disorder until after a constipated habit was fairly established. Usually there is a history of more or less indigestion, both gastric and intestinal, fermentative in character, with acid eructations and flatus, headache, vertigo, palpitation of the heart, nervous irritability, pain at times in the epigastric region, diminished flow of bile, loss of memory, despondency, loss of physical vigor, inability to work without great effort and fatigue, and the general train of symptoms coincident with general ill-health.

The degree in which these symptoms are manifested marks pretty clearly the extent of the disorder. The mouth conditions are those which are characteristic of typical phagedenic pericementitis. The factor of constipation in these cases is important, and should be eliminated as quickly as possible. The rationale of the pathology is within certain limits sufficiently clear. The waste products of nutrition and food *débris* carried off by the intestinal canal are in themselves poisonous to the human organism if resorbed into the system. Add to this source of toxæmia the further factor of putrefaction of the intestinal contents through the agency of micro-organisms usually present, and the toxicity of the mass is enormously increased. Retained within the canal these poisons exert a paralyzing action upon the cells of the intestinal walls and thus destroy peristaltic action, so that artificial means for evacuating the intestine becomes necessary.

As intestinal toxines produce paralysis of peristaltic action, so in an analogous manner do they act as protoplasmic poisons to other tissues to which they may be carried by the blood. It is in this way that they become factors in the irritation of the peridental membrane along with the other waste products of nutrition and of faulty metabolism.

The influence of food habit in this class of cases is of prime importance. Faulty metabolism may be found in the plethoric as well as the anæmic type of individual, and a dietetic scheme which would operate favorably in the one class would be unsuitable to the other. Personally I believe it to be a mistake to interdict the use of red meats and other staple forms of nitrogenous diet as a routine measure in all cases. A liberal allowance of meat in anæmic cases is of positive value.

Carbohydrate foods, especially as sugar, should be avoided or sparingly used, the main principle being to give a nutritious, easily digestible diet regimen, and one not easily fermentable. The treatment of the constipated habit should not include cathartics, but laxative foods, bran bread, fruit, etc., with occasional small doses of Hunyadi water or sodium phosphate if occasion demands an aperient. The use of lithium salt is desirable and the bitartrate is of the highest efficiency in its class of compounds. Liberal use of water, at least two quarts *per diem,* is a valuable aid in washing out the waste products from the system. A thorough hygienic regimen, including all factors conducive to a return to normal health, should be rigidly enforced. All wasteful expenditure of nervous energy is to be avoided; exercise, bathing, and sleep are to be utilized in their proper degree and place for their value in restoring the vital potential of the tissues and general bodily vigor.

I have merely indicated some of the more important lines of treatment, which must be modified or amplified to meet the needs of individual cases. The selective feature which these toxines of faulty metabolism seem to show for the ligamentous structures and particularly for the peridental membrane is noticeable and interesting. It has been suggested, with apparently some justice, that as these dense fibrous structures have relatively less vascularity than many other tissues, and as they are required to perform a relatively large amount of mechanical work, their resistance to toxic irritants is proportionally less than those tissues having a more generous vascular supply and which are well supplied with lymphatics. That the vital resistance of the peridental membrane is of a relatively low grade is shown by the readiness with which those teeth which are subjected to undue strain succumb to pyorrhœa when the tendency is present, as in malocclusion, undue force in wedging, the improper use of ligatures, clamps, etc. The traumatism or strain upon the tissue lessens its vital resistance and makes the membrane of the particular tooth a point of diminished resistance.

I have purposely confined my presentation to but a limited field in order that the protean character of the manifestations of faulty nutrition might not tempt us too far afield in the consideration of what is certainly one of the vitally important problems in our professional work.

After the reading of the paper a number of illustrations of the glandular structures, described by Dr. G. V. Black as existing in the peridental membranes, and others showing the general histological structures of that tissue, were thrown upon the screen and described by the essayist.

